# Explore the Contamination of Antibiotic Resistance Genes (ARGs) and Antibiotic-Resistant Bacteria (ARB) of the Processing Lines at Typical Broiler Slaughterhouse in China

**DOI:** 10.3390/foods14061047

**Published:** 2025-03-19

**Authors:** Lu Ren, Ying Li, Ziyu Ye, Xixi Wang, Xuegang Luo, Fuping Lu, Huabing Zhao

**Affiliations:** 1 Key Laboratory of Industrial Fermentation Microbiology of the Ministry of Education and Tianjin Key Laboratory of Industrial Microbiology, College of Biotechnology, Tianjin University of Science and Technology, Tianjin 300457, China; renlu@mail.tust.edu.cn (L.R.);; 2 China Animal Disease Control Center, Slaughtering Technology Center, Ministry of Agriculture and Rural Affairs, Beijing 102600, China; liyinglucky2001@aliyun.com (Y.L.);

**Keywords:** antibiotic resistance genes, antibiotic-resistant bacteria, broiler slaughterhouse, processing lines, bacterial community, food safety

## Abstract

Farms are a major source of antibiotic resistance genes (ARGs) and antibiotic-resistant bacteria (ARB), and previous research mainly focuses on polluted soils and breeding environments. However, slaughtering is an important link in the transmission of ARGs and ARB from farmland to dining table. In this study, we aim to reveal the pollution of ARGs and ARB in the slaughter process of broilers. First, by qualitative and quantitative analysis of ARGs in samples collected from the broiler slaughtering and processing production chain, the contamination level of ARGs was reflected; secondly, potential hosts for ARGs and microbial community were analyzed to reflect the possible transmission rules; thirdly, through the antibiotic susceptibility spectrum analysis of four typical food-borne pathogens, the distribution of ARB was revealed. The results showed that 24 types of ARGs were detected positive on the broiler slaughter production line, and tetracycline-resistance genes (20.45%) were the most frequently detected. The types of ARGs vary with sampling process, and all sampling links contain high levels of *sul2* and *intI1*. The most abundant ARGs were detected in chicken surface in the scalding stage and entrails surface in the evisceration stage. There was a significant correlation between *intI1* and *tetM*, suggesting that *tetM* might be able to enter the human food chain through class-1 integrons. The host range of the *oqxB* gene is the most extensive, including *Sphingobacterium*, *Bacteroidia unclassified*, *Rothia*, *Microbacterium*, *Algoriella*, etc. In the relevant links of the slaughter production line, the microbial community structure is similar. Removing viscera may cause diffusion of ARGs carried by intestinal microorganisms and contaminate chicken and following processing production. The four food-borne pathogens we tested are widely present in all aspects of the slaughter process, and most of them have multi-drug resistance and even have a high degree of resistance to some veterinary drugs banned by the Ministry of Agriculture. Our study preliminarily revealed the pollution of ARGs and ARB in the slaughter process of broilers, and these results are helpful to carry out food safety risk assessment and formulate corresponding control measures.

## 1. Introduction

Antimicrobial resistance (AMR) has become a major public health crisis in recent years. It is estimated that deaths caused by AMR could climb from 700,000 annually in 2014 to 10 million by 2050 [1]. The use, misuse and abuse of antibiotics in veterinary, agriculture and clinical therapy for decades have increased the prevalence of antibiotics resistance genes (ARGs), especially the acquired resistance elements by horizontal gene transfer into the human and animal microbiomes [2]. ARGs can spread among the same or different species of bacteria through various molecular mechanisms, such as integrons, plasmids, transposons, etc., resulting in the emergence and rapid spread of antibiotic resistant bacteria (ARB) [3]. The long-term use of low-dose antimicrobials as growth promoters in animal production is still a common practice in many countries, which increases the economic benefits but also hides a great risk that the continuous use of antimicrobials promotes and exacerbates the emergence of drug-resistant bacteria [4,5]. Evaluating the impact of antimicrobial use in animal production on bacterial resistance in animals, humans and the environment remains a great challenge due to the interdependence and close interrelationship between humans, animals, and the environment [6,7]. Antibiotic abuse, misuse, or inappropriate prescribing can lead to the spread of AMR infections caused by pathogens that are first susceptible to antibiotics, after which the bacteria are able to acquire ARGs and act through a variety of mechanisms [8]. Bacteria with ARGs on transferable genetic elements have been identified in many microbial communities, including those associated with humans and countless communities associated with animals [9]. Unconventional use of antibiotics on food animal farms can accelerate the development and spread of bacterial resistance in ARB and ARGs, posing an increasing threat to human and animal health [10,11,12]. Farmed animals are an important source of antibiotic-resistant bacteria, and consumption of animal products has been associated with an increased risk of certain antibiotic-resistant infections [13,14,15].

Despite the increasing concerns over inappropriate use of antibiotics in veterinary medicine and food production, slaughterhouse and meat products remain potential reservoirs of ARGs and ARBs [16]. The prolonged and intensive use of antibiotics in food animal husbandry may lead to the accumulation of antibiotics in animal guts and the receiving environment, as well as sustained selective pressure on ARGs and ARBs [17,18]. ARGs in farm environments are widely distributed in soil, water, manure, and animal feces, primarily driven by factors such as antibiotic use, animal density, and waste management practices. The ARGs originating from farms can subsequently enter the food chain, particularly during animal transportation and processing in slaughterhouses. In slaughterhouse environments, ARGs are frequently detected in wastewater, surfaces, and animal carcasses, where their prevalence is often amplified due to the high concentration of microorganisms and the intensive processing activities. At the same time, factors such as contaminant exposure and horizontal genetic transfer (HGT) increase the risk of transmission of ARGs and ARBs from animal farms to the natural environment and humans [19]. This makes slaughterhouses a critical point for ARG dissemination and a key focus for research, as they represent a bridge between farm environments and human exposure through food products. Consequently, ARGs and ARB can spread to humans throughout the food-supply chain by exposure via contaminated animals, meat products, or natural environment (i.e., air, water, and soil) [20,21,22]. Unconventional use of antibiotics on food animal farms can accelerate the development and spread of bacterial resistance in ARB and ARGs, posing an increasing threat to human and animal health [23].

With the increase in global consumption of poultry meat, bacterial pathogens act as important factors affecting the safety of poultry and raw meat [24]. In recent years, the production and sales of chicken have shown rapid and steady growth in the world. According to the United States Department of Agriculture, world chicken production has maintained steady growth, increasing from 92.47 million tons in 2018 to 102.389 million tons in 2023 [25]. China is the world’s third largest producer of broiler chickens, with production reaching 14.30 million tons in 2023 [26]. Chicken meat products are rich in nutrients, as well as cross-contamination generated during processing in slaughterhouses; fresh chicken meat is susceptible to contamination by food-borne microorganisms and cross-contamination between carcasses, environmental hygiene, utensils, and staff operations during slaughter and processing increases the level of microbial contamination on the surface of carcasses, which directly affects the safety and hygiene of chicken meat [27,28,29]. Therefore, broiler slaughtering is one of the important links in the prevention and control of ARGs and ARB diffusion.

Most of the previous studies focused on the surrounding environment of the broiler slaughterhouse [30]. Actually, the slaughtering process could play crucial roles in the transmission of ARGs and ARB to humans via environmental interfaces and meat products. Bleeding, evisceration, and other related processes can contaminate broiler carcasses and equipment, leading to the spread of gut bacteria [31]. The gut microbiome often becomes particularly problematic since they represent a complex ecosystem and a epicenter of horizontally transferrable resistance traits between commensals and pathogens, and they cross-transmit resistant strains between animals and humans. Antibiotic resistance can be transmitted through vertical gene transfer (VGT) and HGT [32,33], and animal feces is an important reservoir and source of ARGs in the environment, creating favorable conditions for HGT events. HGT can be achieved by conjugation, transduction, and transformation [34]. In addition, these resistant bacteria continue to exist in the environment even after antibiotic selective pressure has been eliminated, resulting in persistent contamination effects [35]. Therefore, it is crucial to prioritize the exploration of the risk of antibiotic resistance distribution during slaughter.

Many studies have focused on MDR foodborne pathogens and commensal bacteria, mainly occurring in food and the food chain [31,36]. These results indicate that respective intervention measures along the slaughter processing line should aim at reducing the microbiological load on broiler carcasses as well as preventing cross-contamination. However, the data are insufficient on ARGs and ARB contamination in various stages of the processing lines of broiler slaughterhouse. In this study, we aimed to investigate the pollution of ARGs and ARBs in each key stages of the typical broiler slaughter production chain in order to achieve guidance on risk assessment in broiler slaughtering and processing in the future.

## 2. Materials and Methods

### 2.1. Sample Collection

**Figure 1:**
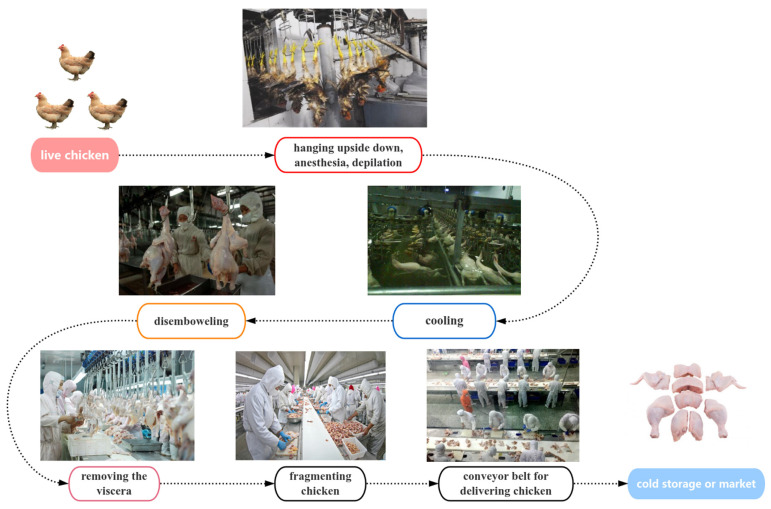
Broiler slaughter process and location distribution of sampling sites. (Pictures were collected from http://image.baidu.com; accessed on 21 September 2024).

The samples of different stages of the broiler production processing were collected from a typical broilers slaughterhouse in Liaoning Dandong, China (Longitude 124°23′ E, Latitude 40°07′ N), which receives animals from multiple suppliers and all over the country. A total of 205 samples were collected. Ten representative links in the broiler slaughtering and processing chain were selected for sample collection, and the whole broiler slaughtering and processing chain was divided into the hanging link, electric anesthesia stabbing link, blood draining link, scalded link, pre-cooling link, dehairing and finishing link, offal removal link, splitting link, and packaging link. The selected representative links are scalded, cleaned, gutted, and split. A schematic diagram of the broiler-slaughtering and -processing production chain is shown in Figure 1. To ensure reproducibility and minimize contamination during sample collection and processing, strict quality control measures were implemented. Samples were collected under aseptic conditions in accordance with national standards (GB 4789.1-2016, GB/T 19480, and NY/T 541-2016). Sam pling personnel were trained in sterile techniques and wore protective equipment (e.g., gloves, masks, and sterile clothing) to prevent contamination. 

With reference to GB/T 4789.17-2003’s non-destructive sampling methods, according to the special sampling conditions in the slaughtering production chain using 3M’s special sampling applicator stick, the head of the stick is aseptic sponges, and the head of the sponge is placed in 10 mL sterilized buffered peptone water (BPW) to seal [37]. In accordance with the stipulations of SN/T 4092-2015, “Carcass Sampling Methods for Microbiological Testing”, the sponge swab affixed to the sealed applicator stick was initially saturated with sterilized BPW. Subsequently, the designated specification plate was firmly pressed against the surface of the selected carcass. The sponge swab was then meticulously rubbed across the entire area delineated by the specification plate (measuring 10 cm by 10 cm), ensuring comprehensive coverage. This was achieved by maneuvering the swab in a horizontal motion, followed by flipping the sterilized sponge swab to guarantee that each facet of the swab made complete contact with the sampled carcass surface. Each side of the swab can fully contact the sampling surface, and the time of application of the wipe is controlled for a uniform time. The collection of clean cooling-water samples is conducted in the same way in the water and on the drainage surface; it is performed a certain period of time after the completion of the wipe sampling and is immediately placed in the preservation solution of the sterilized BPW stick, which is stored for preservation in a tube, which was screwed tightly and marked well. The collected samples were transported back to the laboratory at 4 °C.

The sampled swabs were fully shaken in the BPW preservation solution for about 5 min, and all the preservation solution after shaking was transferred to a sterilized 50 mL centrifuge tube and then centrifuged at a low speed of 1000 r/min for 15 min; then, the supernatant was poured off, and the precipitate was taken to extract the total genomic DNA of the samples.

**Figure 2:**
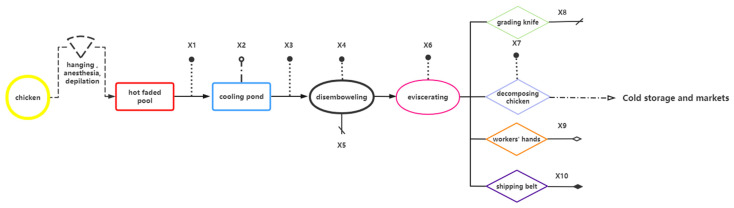
Simplified diagram of the broiler-slaughtering and -processing production chain and sampling points. Note: The dotted line represents meat sampled, the dotted solid line represents water sampled, the solid line with a slash represents the surface of a knife sampled, the solid line with a hollow rhombus represents the surface of an operator’s hand sampled, and the solid line with a solid rhombus represents the surface of a conveyor belt sampled.

The schematic diagram of the distribution of the ten sampling points and the number of samples collected at each point are illustrated in Figure 2 and Table 1.

### 2.2. DNA Extraction of Samples

Genomic DNA was extracted with TIANamp Micro DNA Kit (TianGen, Beijing, China) according to the manufacturer’s instruction. The concentrations and qualities of DNA extracted were determined with a NanoDrop ND-2000 (Thermo Scientific, Waltham, MA, USA). The DNA samples were stored at −20 °C until further analysis.

### 2.3. Qualitative and Quantitative PCR of ARGs and intI 1

Forty-three ARGs and a class I integron gene (*intI 1*) were tested via a PCR assay. The primers used are listed in Table 2. Furthermore, 11 ARGs (*tetM*, *tetX*, *tetT*, *tetQ*, *sul2*, *mefA*, *bla_TEM_*, *floR*, *oqxB*, *aadA1*, *aac(6’)-ib-cr*) and *intI 1*, as well as the 16S rRNA encoding gene, were quantified using the real-time PCR on an ABI StepOnePlus instrument (Applied Bio-systems, Foster City, CA, USA). The abundance of the above-listed genes was quantified via qPCR, targeting different fragments of ARGs that were cloned into plasmid vectors (pBackZero-T vector, Takara, Kyoto, Japan) and used as standards. A total of 2 mg of RNA was converted to cDNA using LunaScript RT SuperMix Kit (NEB, Ipswich, MA, USA) as per the manufacturer’s guidelines [38]. Gene expression analysis was performed by conducting RT-PCR with the SYBR Green Premix Pro Taq HS qPCR kit from Accurate Biotechnology in China. All gene expression levels were normalized to the levels of 16S rDNA. All primers used in this study were synthesized by GENEWIZ Biotechnology Co. (Tianjin, China). The relative mRNA levels of the PCR products were quantified using 16S rDNA as an internal standard and calculated with the 2^−ΔΔCt^ method.

### 2.4. Microbial Community Analysis

High-throughput sequencing of the 16S rRNA gene was conducted by Shanghai Whaleboat Gene Technology Co., Ltd. (Shanghai, China) using the Illumina HiSeq platform. The variable V3 and V4 regions of the bacterial 16S rRNA gene were amplified using the primer pair 341F (5′-CCTAYGGGRBGCASCAG-3′) and 806R (5′-GGACTACHVGGGTWTCTAAT-3′). Raw sequencing data underwent a quality control process using UPARSE (version 7.0.1090). The PCR products were detected by agarose gel electrophoresis and cut and recovered for purification. The purified PCR products were subjected to secondary PCR and, at the same time, connected to the Hiseq2500 PE250 sequencing connector (Illumina, San Diego, CA, USA), and sequencing libraries were constructed for sequencing. Finally, clustering and species classification analyses were performed based on the effective sequencing results in terms of Operational Taxonomic Units (OTUs), α-diversity analysis, β-diversity analysis, Hierarchical clustering (Hierarchical clustering), Principal Coordinate Analysis (PCoA), and species difference analysis. Classification and abundance visualization were performed using Greengene database, Krona software package, etc. The analysis and visualization were performed using data-processing tools, including Origin 2017 and R (version 3.3.1). All raw sequence datasets obtained in this study have been deposited in the NCBI Sequence Read Archive (SRA) under the BioProject accession number PRJNA1218474.

### 2.5. Screening and Antibiotic Resistance Test of Foodborne Pathogens

From a total of 250 samples collected from the broiler slaughter and processing chain, foodborne pathogens, *Listeria monocytogenes*, *Salmonella*, *Staphylococcus aureus*, and *Escherichia coli*, were, respectively, screened according to National Standard of the People’s Republic of China GB 4789.30-2016, GB 4789.4-2016, GB 4789.10-2016 [39,40,41].

*Escherichia coli* (*E. coli*): The isolation and identification of *E. coli* were performed according to the national standard method GB 4789.30-2016. Samples were aseptically inoculated onto MacConkey and Erythromycin agar plates and incubated overnight. Typical colonies were selected and purified by streaking on the MacConkey agar. After a third purification, the colonies were inoculated onto a nutrient agar and incubated at 37 °C overnight. Suspected E. coli colonies were subjected to Gram staining and microscopic examination to confirm staining characteristics.

*Salmonella*: Samples were pre-enriched in BPW for 8 h, followed by enrichment in TTB (1:10 ratio) at 42 °C and SC (1:10 ratio) at 37 °C. Enriched cultures were streaked onto XLD and BS agar plates. XLD plates were incubated at 37 °C for 24 h, while BS plates were incubated at 37 °C for 48 h. Suspected colonies on the XLD agar appeared pink (with or without black centers), yellow, or as large black colonies. On the BS agar, the colonies were black with a metallic luster, brown, gray, or gray–green, with surrounding media potentially turning black, brown, or unchanged. Suspected Salmonella colonies were inoculated onto TSI slants and incubated at 37 °C for 24 h. Cultures exhibiting Salmonella-typical TSI biochemical characteristics were selected for PCR identification.

*Single-enriched Listeria monocytogenes* (*L. monocytogenes*): The isolation and identification of *L. monocytogenes* were conducted according to the national standard method GB 4789.30-2016. Aseptically, 25 g (mL) of the sample was homogenized with 225 mL of LB1 enrichment broth (1:10 ratio) and incubated at 30 ± 1 °C for 24 h. Subsequently, 0.1 mL of the enriched culture was transferred to 10 mL of LB2 enrichment broth and incubated at 30 °C for an additional 24 h. The LB2 culture was streaked onto PALCAM agar or *L. monocytogenes* chromogenic plates and incubated at 36 °C for 24–48 h. Typical or suspicious colonies were selected and inoculated into rhamnose and xylose fermentation tubes, followed by incubation at 36 ± 1 °C for 24 h. Simultaneously, colonies were streaked onto TSA-YE plates for purification and incubated at 36 ± 1 °C for 36 h. Rhamnose-positive and xylose-negative colonies were further purified and identified.

*Campylobacter jejuni* (*C. jejuni*): The swabs were vigorously mixed in BPW preservation solution, followed by low-speed centrifugation at 4000 r/min for 30 min. The supernatant was discarded, and the pellet was resuspended in 10 mL of Brinell’s broth (supplemented with additives and horse serum), then transferred to 90 mL of nutrient broth. The broth was placed in an anaerobic jar with microaerobic gas packs and indicators, creating a microaerobic environment (5% O_2_, 10% CO_2_, 85% N_2_), and incubated at 42 °C for 48 h. The enriched culture was streaked onto modified Skirrow’s agar plates and incubated under microaerobic conditions for 48 h. Single colonies were then streaked onto Columbia blood agar plates (containing 5% defibrinated sheep blood) for further cultivation.

*Staphylococcus aureus*: The sponge swab was vigorously shaken in the BPW preservation solution, and 1.5 mL of the solution was transferred into 10 mL of 7.5% NaCl nutrient broth, followed by incubation at 37 °C for 24 h. This process was repeated twice, with the final incubation lasting 20 min. The enriched culture was then streaked onto sheep blood agar plates and incubated at 37 °C for 24 h. Colonies exhibiting opaque, moist, glossy surfaces (yellow or white) with surrounding transparent hemolysis rings were selected for purification. Purified colonies were Gram-stained and examined microscopically for further identification.

Detection of various drugs commonly used in China’s poultry industry, such as macrolides, β-lactams, colchicine, aminoglycosides, aminoglycosides, aminoglycosides, and alcohols, as well as the current regulations of the Ministry of Agriculture, can no longer be used as veterinary therapeutic drugs. The broth dilution method was used to test a total of 26 antibiotics, including penicillin, ouaghmentine, erythromycin, clindamycin, enrofloxacin, ofloxacin, cefotifur, cefoxitin, sulphonylisoxazole, vancomycin, compound Neonormin, Doxycycline, Tamiflu, Fosfenicol, Tilmicin, Gentamicin, Linezolid, Ampicillin, Augmentin, Spectinomycin, Ceftazidime, Meropenem, Ampramycin, Mucomycin, Acetylmethoquine, and Tetracycline. The Minimum Inhibitory Concentration (MIC) was interpreted according to the guidelines of the Clinical and Laboratory Standards Institute (CLSI). Moreover, based on the corresponding standards of the American CLSI, the corresponding results of sensitive (S), moderately sensitive (I), and drug-resistant (R) were obtained.

### 2.6. Data Analysis and Visualization

Quantitative information was presented as the average plus the standard error of the average (SEM). SPSS Statistics 23.0 was utilized to analyze the data through a one-way analysis of variance. Network analysis was conducted using R3.3.1 and the Gephi (0.9.2) platform.

## 3. Results and Discussion

### 3.1. Pollution of ARGs

**Figure 3:**
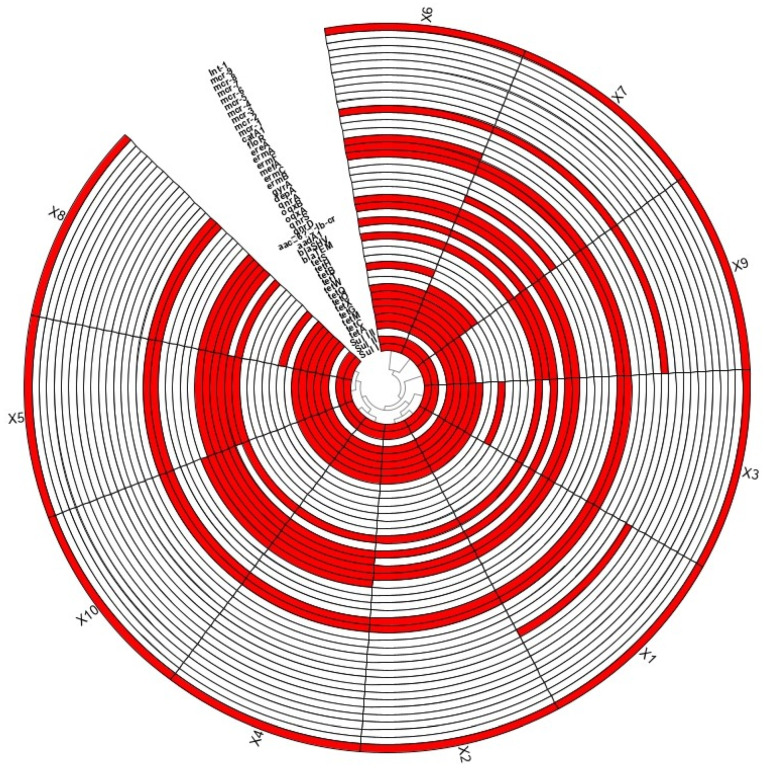
Qualitative test results of ARGs at ten sampling points in the broiler slaughter and processing chain. Note: Red represents the target gene detected positively, white represents the target gene detected negatively; X1: carcasses surface, X2: cooling pool water, X3: carcasses surface after washing with cooling water, X4: carcasses surface, X5: knife surface, X6: entrails surface, X7: carcasses surface, X8: knife surface, X9: hand surface, X10: conveyor surface.

According to the quantitative results of ARGs and *intI1* (Figure 3), qualitative testing across eight major antibiotic types revealed the presence of ARGs in each category. Most ARGs were consistently detected across all sampling sessions, with *tetQ*, *tetO*, and *tetW* being particularly prominent. In contrast, *blaSHV* and *oqxA* were absent in most sessions and only detected in two instances, while *tetT*, *mefA*, and *oqxB* appeared in isolated sessions. Notably, *intI1*, a key integron gene facilitating ARGs transfer, was detected in all 10 sampling sessions.

Forty-three ARGs covering eight major types of antibiotic resistance and *intI1* were tested in samples from broiler slaughter/processing chain, and twenty-three ARGs and *intI1* were found to be positive. The ratio of detected links to ten total links is counted as the detection rate. Tetracycline-resistance genes (20.45%) were the most frequently detected, followed by macrolide resistance genes (6.82%) and quinolones resistance genes (6.82%), sulfonamide resistance genes (4.55%), β-lactam resistance genes (4.55%), chloramphenicol resistance genes (2.27%), and class I integron genes (2.27%), while peptide resistance genes were not detected. The kinds of ARGs detected in samples from each sampling points (X1–X10) are summarized in Figure 3. The highest diversity of ARGs was detected in sampling points X6 and X7 with a total of 18 kinds of ARGs; followed by X4, X5, X8, and X10 with sixteen kinds of ARGs; X1, X3, and X9 with fifteen kinds of ARGs; and X2 with fourteen kinds of ARGs. ARG spectrums for sampling points X1 and X2, X4 and X10, X5 and X8, X6 and X7 were similar. All parts of the slaughter chain were detected with positive ARGs, which suggested that the contamination of ARGs in the slaughter process of broilers was widespread.

Based on the qualitative results, ARGs heatmaps were produced, from which it can be seen clearly and intuitively that, among the forty-four ARGs and *intI 1* detected qualitatively, the highest number of ARGs and *intI 1* was detected on the surface of the X6 viscera and the surface of the meat in the X7 splitting session, with eighteen detected, followed by the surface of the X10 splitting conveyor belt, with seventeen; the same number of detections was found on the surfaces of the knives of the X5 clearing session and the knives of the X8 splitting session; the same number of detections was found on the surfaces of the knives of the X8 splitting session. Surface had the same number of detections at sixteen; X1 blanch fading session meat surface, X4 clean-ripping session meat surface, and X9 segmentation session worker’s hand surface had fifteen detections. X2 cooling water had fourteen detections, and X3 cooling session meat surface had the lowest number of detections, but the number of detections was as high as thirteen.

The clustering information shown in the heatmap indicated that the detection of ARGs and *intI 1* were similar for X1 blanching and fading session meat surfaces and X2 cooling-session water, the detections of ARGs and *intI 1* were similar for X4 clean-gutted-session meat surfaces and X10 segmentation session conveyor belt surfaces, and the similarity of detection of ARGs and *intI 1* was high for X5 clean-gutted-session knife surfaces and X8 segmentation session knife surfaces. The detection of ARGs and *intI 1* on the surface of the X6 offal and the surface of the meat in the X7 splitting session after removal of the offal were similar. X1 (scalding) and X6 (visceral removal) had the highest proportion of drug-resistant genes. X1 was the surface sample of chicken after scalding and alopecia, and the main drug-resistant gene was *intI 1*, indicating that broilers entering the slaughtering and processing chain from the outside would bring drug-resistant genes from the skin, feathers, or external environment. X6 is the sample collected during viscera extraction, and the main drug resistance gene is sul2, indicating that ARGs is likely to be contaminated by intestinal microorganisms due to viscera extraction.

**Figure 4:**
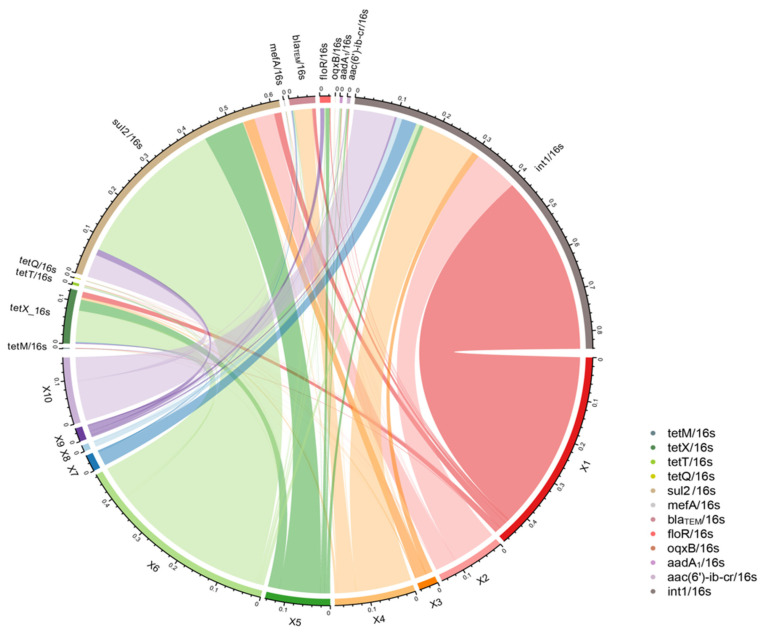
Types of ARGs and their abundance distribution (visualized by Circos) from ten sampling points in the broiler slaughter processing chain. Note: The length of the outer ring bar represents the percentage of ARGs in each sample. Each ARG and sampling point is represented by a specific ribbon color, and the width of each ribbon indicates the abundance of ARGs. X1: carcasses surface, X2: cooling pool water, X3: carcasses surface after washing with cooling water, X4: carcasses surface, X5: knife surface, X6: entrails surface, X7: carcasses surface, X8: knife surface, X9: hand surface, X10: conveyor surface.

Figure 4 illustrates which ARGs contribute most to these samples, and which sampling points they are distributed. The most abundant ARGs were detected in the samples from X1 and X6, followed by the sample from X4, X2, X5, and X10. A relatively less abundance of ARGs was detected in the samples from X3, X7, X8, X9. In general, *intI1*, *sul2*, *tetX*, *bla_TEM_*, and *floR* have the highest pollution levels, and *tetM*, *tetT*, *tetQ*, *mefA*, and *oqxB* have the lowest pollution levels. The most abundant *intI1* is mainly derived from X1, X4, X2, and X10, and the second-highest abundance of *sul2* is mainly derived from X6 and X5. The combined analysis of Figure 3 and Figure 4 indicates that the abundance of *intI1* and *sul2* is very high in the entire sampling chain. According to the analysis of qualitative results, eleven ARGs and *intI 1*, involving the eight major types of antibiotics that have research and analysis value, were selected for the quantitative analysis. The highest abundance levels of ARGs and *intI 1* were detected on the meat surface of X1 scalded and faded session and on the surface of the viscera of X6. *intI 1* abundance was very high in X1 (a sample of the chicken meat surface collected after scalded and dehairing). X6 was a sample collected during removal of viscera by rifling, and the ARGs with higher abundance were *sul2* (0.446958626); most of *sul2* was detected in chicken intestines and chicken feces in previous studies, so *sul2* suggests to us that ARGs in this chain may originate from intestinal microbial contamination due to manipulation at the time of removal of viscera. The main types of ARGs causing contamination in the broiler slaughter chain were *sul2*, *tetX*, *floR*, and *oqxB*, whereas *intI 1* was detected with a high detection rate and abundance, suggesting that the detected ARGs have a high risk of transmission.

### 3.2. Transmission Patterns of Antibiotic Resistance Genes in the Production Chain of Broiler Slaughtering and Processing

**Figure 5:**
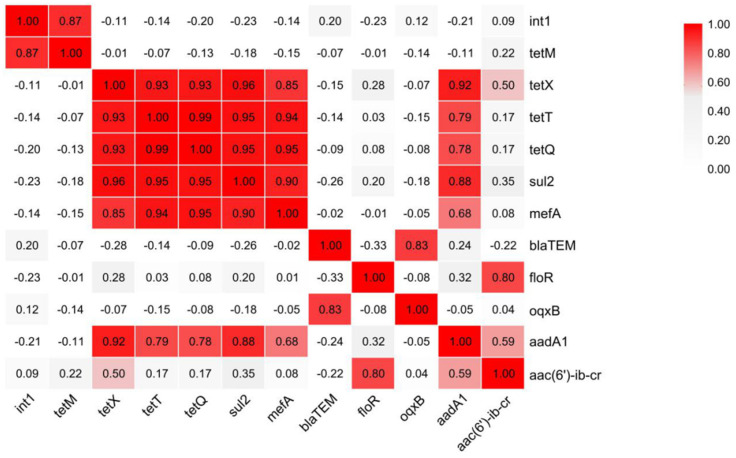
Correlation analysis of twelve quantitatively detected genes.

Among various genetic mechanisms that are involved in the dissemination of ARGs, integrons play a vital role [42]. Figure 5 demonstrated that there is a significant correlation between *intI1* and *tetM*, which suggested *tetM* might have the potential to enter the human food chain via the Class-1 integrons. In addition, there is also a significant correlation between *tetX*, *tetT*, *tetQ*, *sul2*, *mefA*, and *aadA1*; a significant correlation between *floR* and *aac(6′)-ib-cr*; and a significant correlation between *mefA* and *aadA1*, which suggested that these ARGs have a joint relationship during diffusion.

A correlation analysis of the twelve quantitatively detected genes showed significant correlations greater than 0.7 at the Pearson 0.01 level and greater than 0.5 and less than 0.7 at the Pearson 0.05 level. The prevalence of significant correlations between ARGs and the type I integron gene *intI 1* suggests that the ARGs detected in our broiler slaughter and processing chain have the ability to transfer horizontally.

### 3.3. Correlation Analysis of Microbial Community at Different Slaughtering Links

**Figure 6:**
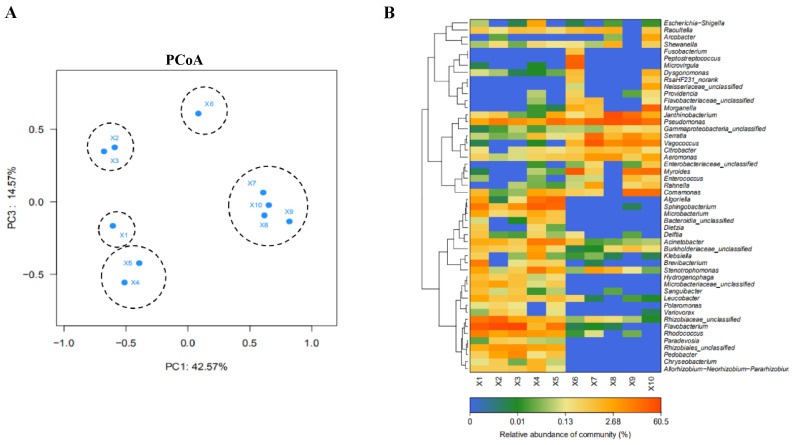
(**A**) PCoA analysis shows correlation between ten sampling points in the broiler slaughter and processing chain. (**B**) Heat map analysis of the most common genera in samples from ten sampling points on the broiler slaughter and processing process. Clustering using Spearman rank correlation. Different colors indicate the relative abundance of each genus. The sample name indicates the sampling location. Note: X1: carcasses surface, X2: cooling pool water, X3: carcasses surface after washing with cooling water, X4: carcasses surface, X5: knife surface, X6: entrails surface, X7: carcasses surface, X8: knife surface, X9: hand surface, X10: conveyor surface.

The results of the PCoA (Figure 6A) showed that the community compositions in the slaughtering process were clustered into five groups: ironing (X1), cooling (X2, X3), eviscerating (X4, X5), removing viscera (X6), and segmenting (X7–X10). This result further indicates that the community composition of adjacent operation links was similar. X1 and X6 have unique community composition and differ significantly from the other links.

Based on the heat map results of the community analysis, the main microbial components were *Pseudomonadaceae*, *Serrateae*, *Vaginococcus*, *Citrobacter*, *Aeromonas*, *Burkholderia* and *Larnella*, among others. By analyzing the structure of the microbial community (Figure 6B), it is clear that X1 and X6 are representative of the structure of the two major groups of microbiomes or the starting point of contamination: the dominant microbial community in the X1 hot fading link was *Rhizobium*, *Staphylococcus aureus*, *Rhizobium landis*, *Rhodococcus* and *Flavobacterium*; these genera were mainly from the environment, suggesting that the microbial contamination in this link mainly came from the breeding environment; the structure of microbial community was significantly changed after X6, which mainly included *Pseudomonadaceae*, *Serrateae*, *Vaginococcus*, *Citrobacter*, *Aeromonas*, *Burkholderia* and *Larnella*, which are mainly of enteric origin. X6 net chamber session resulted in leakage of enteric microorganisms contaminating the subsequent sampling session. Meanwhile, Figure 6B showed that the microbial community structure was well correlated in the sampling session. The abundance of these bacteria could be effectively reduced by removing the division stage of the gut organs. *Flavobacterium*, *Pseudomonadaceae* and *Sphingomonas* were the main bacteria in chicken feces, and these bacteria may be carried into the breeding environment [43]. In addition, due to its low relative abundance, it is able to colonize retail-packaged meat kept at refrigerated temperatures, raising food safety concerns [44].

ARGs can persist in extracellular gene elements, such as plasmids and even naked DNA fragments, but their replications need to proceed intracellularly [45]. Thus, the change in bacterial communities is prone to affect the proliferation and behavior of ARGs [46]. The heat map results of the community analysis clearly showed the differences before and after sampling point X6. Microbial communities from sampling points X1-X5 clustered together, and high-abundance genera mainly include *Acinetobacter*, *Stenotrophomonas*, *Rhizobiaceae-unclassified*, *Flavobacterium*, *Rhodococcus*, etc., mainly belonging to environmental microorganisms. Meanwhile, microbial communities from sampling points X6–X10 clustered together, and the high-abundance genera mainly include *Pseudodomonas*, *Serratia*, *Vagococcus*, *Citrobacter*, *Aeromonas*, *Burkholderiaceae*, *Janthinobacterium* and *Rahnella*, etc., mainly belonging to intestinal microorganisms. It might be inferred that the X1 and follow-up links are polluted by bacteria from fur and breeding environments, while the X6 and follow-up links are contaminated by bacteria from internal organs. The ARGs that cause the contamination of the broiler chain mainly include *intI 1*, *sul2*, *tetX*, *floR*, *oqxB*, etc. X1 (scalding) and X6 (dirty removal) points caused significant changes in the community, and the community composition of the slaughter-related points was similar.

### 3.4. Analysis of Potential Hosts of ARGs and intI 1

**Figure 7:**
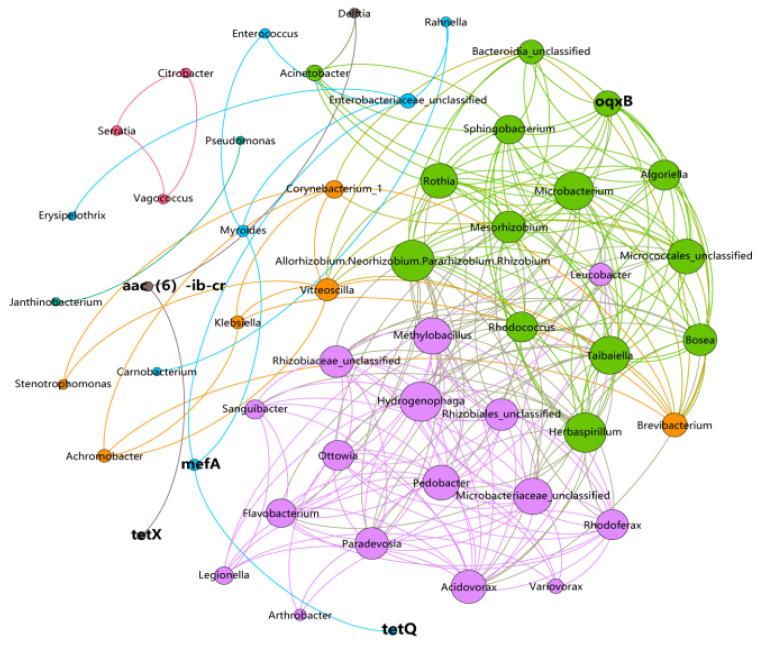
Network analysis of cooccurring ARGs and potential host bacteria (top 30 genera) based on Pearson’s correlation coefficients (*p* < 0.01, r > 0.80). Note: Bold fonts represent antibiotic resistance genes, and the nodes with different colors represent the related bacteria genus. The node size is proportional to the number of connections (degree). An edge represents a significant and strong correlation, where the edge thickness is proportional to the Pearson’s correlation coefficient.

Bacteria are the main carriers of resistance genes [47], and the correlation between genera with high abundance and ARGs was investigated using network analysis to identify potential host bacteria for ARGs. As shown in Figure 7, the correlation between genera with high abundance and ARGs was studied via network analysis to identify potential ARGs host bacteria. In total, 53 nodes and 255 edges were obtained. These edges indicated significant correlations (*p* < 0.01 and r > 0.80) between ARGs, between bacterial communities, and between ARGs and bacterial communities across the ten sampling sites, with a total of fifteen potential hosts of ARGs identified. *oqxB*’s potential host bacteria belonged to the *Enterobacteriaceae*, *Rhizobia rhizobia*, and *Microbacteria Micro bacteria*. *Algle*, *Enterobacteriaceae*, and *Fragrance* species were the potential host bacteria of *mefA*. *aac(6)-ib-cr* in *Delftia* was the only potential host bacteria. Network analyses also showed significant correlations (*p* < 0.01) between *aac(6′)-ib-cr* and *tetX* and between *tetQ* and *mefA*, but their potential host bacteria had lower abundance.

The correlations between the top bacterial genera and ARGs were studied with a network analysis in order to identify the potential host bacteria for ARGs. As shown in Figure 7, 53 nodes and 255 edges were obtained. These edges indicated that there were significant correlations between ARGs, between bacterial communities and between ARGs and bacterial communities in the ten sample points (*p* < 0.01 and r > 0.80). Fifteen potential hosts of ARGs were identified among the top 100 genera. The potential host bacteria for *oqxB* belonged to the *Methylobacillus Acinetobacter*, *Enterobacteriaceae unclassified* and *Allorhizobium*. *Neorhizobium*, *Pararhizobium*, *Rhizobium*, *Taibaiella*, *Sphingobacterium*, *Mesorhizobium*, *Bacteroidia unclassified*, *Micrococcales unclassified*, *Bosea Rothia*, *Microbacterium*, *Algoriella*, *Enterobacteriaceae* and *Myroides* are potential host bacteria for *mefA*. The only potential host bacteria for *aac(6′)-ib-cr* is *Delftia*. The network analysis also demonstrated that there were significant correlations between *aac(6′)-ib-cr* and *tetX* and between *tetQ* and *mefA* (*p* < 0.01), but their potential host bacteria did not have high abundances. Due to being influenced by several bacteria within the Enterobacteriaceae, several studies have indicated meat as a reservoir of ARGs and a potential source of the environmental contamination [48]. Because most environmental bacteria are still not culturable using current techniques, only a few ARG hosts were experimentally verified in previous studies [49]. Therefore, the co-occurrence patterns could provide a preliminary, albeit inconclusive, assessment for finding possible ARG hosts in complex environmental samples such as field environments.

### 3.5. Contamination with Food-Borne Antibiotic-Resistant Bacteria in the Production Chain of Broiler Slaughtering and Processing

**Figure 8:**
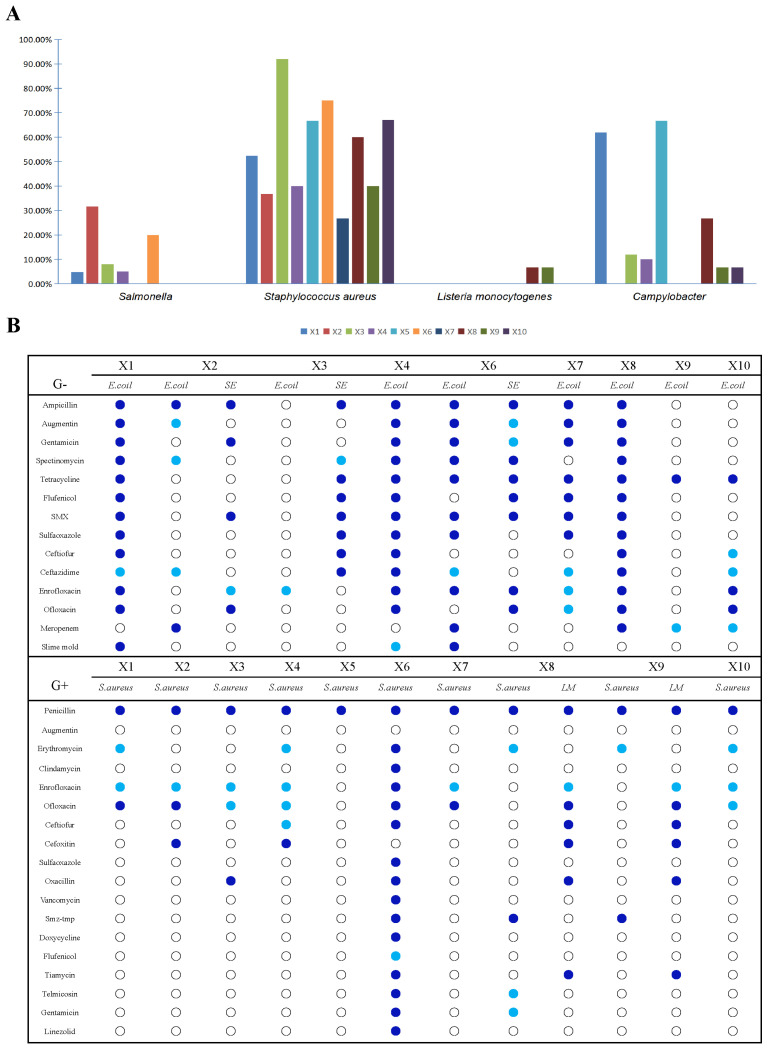
(**A**) Proportion of four foodborne pathogens, *Salmonella*, *Staphylococcus aureus*, *Listeria monocytogenes*, and *Campylobacter jejuni*, detected at ten sampling sites. (**B**) The results of susceptibility tests. Note: *Escherichia coli* (*E. coli*), *Salmonella* (*SE*), *Staphylococcus aureus* (*S. aureus*), and *Listeria monocytogenes* (*LM*) screened at different stages. The test results showed three types of resistance (● dark blue dots), poisoning sensitivity (● light blue dots), and sensitivity (○ white dots); X1: carcasses surface, X2: cooling pool water, X3: carcasses surface after washing with cooling water, X4: carcasses surface, X5: knife surface, X6: entrails surface, X7: carcasses surface, X8: knife surface, X9: hand surface, X10: conveyor surface.

A total of 205 samples were collected from the slaughter and processing production chain of broilers, and five foodborne pathogens were detected against the above samples, namely pathogenic *E. coli*, *S. aureus*, *L. monocytogenes*, *Salmonella*, and *C. jejuni*. The results showed (Figure 8A) that pathogenic *E. coli* were not detected in all samples; *Salmonella* was detected at the highest rate of 31.6% in X2 pre-cooled water; *S. aureus* was detected in all segments, with the highest rate of 92% on the surface of the meat after pre-cooling in X3; *L. monocytogenes* was detected only on the surface of the meat cut in the separator knives X9 and X8, with a detection rate of 6.7%; *C. jejuni* was not detected only in the organ excision section of X6 and in the segmented meat of X7, with the highest detection rate occurring on the surface of the organ meats, with a detection rate of 66.7%.

Typically, *Staphylococcus aureus* was common in the slaughter and processing chain of broilers, with the highest detection rate being in scalding water and Salmonella being detected at the highest rate in the pre-cooling area. Notably, *L. monocytogenes* was detected only during the splitting process. In previous studies, when broilers just entered the processing plant, the detection rate of *Salmonella* was 3–4%, and the positivity rate could reach 20–35% at the end of slaughtering and processing, which indicates that the slaughtering and processing of broilers is an important process in which *Salmonella* contamination occurs [50]. *Salmonella* in broiler slaughtering and processing mainly originates from cross-contamination between carcasses and environmental and equipment contamination, and scalding, depilation, gutting, and cooling are considered to be the main links of cross-contamination of broiler carcasses [51].

Antibiotic drug sensitivity test panels are divided into aerobic Gram-positive and Gram-negative bacterial tests. In this experiment, four aerobic foodborne pathogens with research value were detected: *E. coli* (*virulence factor*), *Staphylococcus aureus*, *Salmonella*, and *L. monocytogenes*, of which *E. coli* and *Salmonella* are Gram-negative, and *L. monocytogenes* and *Staphylococcus aureus* are Gram-positive. Figure 8B shows a drug sensitivity profile showing three results denoted as follows: drug-resistant (dark blue dots), drug moderately sensitive (light blue dots), and drug-sensitive (white dots). The *E. coli* screened at the surface of the scalded meat, at the surface of the clean rifted meat and at the surface of the cutter in the splitting process showed multi-drug resistance and high drug resistance. *Salmonella* spp. screened only in X2 cooled water, X3 cooled meat, and X6 offal removal sessions were also multi-drug-resistant. In the antibiotic sensitivity test, ampicillin, tetracycline, florfenicol, enrofloxacin, ofloxacin, and mucomycin were all banned veterinary drugs by the Ministry of Agriculture, but the results of the sensitivity test showed that the foodborne pathogens detected were still resistant to the antibiotics, and the follow-up sensitivity test for mucomycin revealed that *E. coli* detected at the X6 offal removal session was ultra-highly resistant to mucomycin. Gram-positive bacteria *L. monocytogenes* and *Staphylococcus aureus*, which can be detected at every step of the slaughter and processing chain of broiler chickens, were detected in the X6 removal of viscera link with a very high level of resistance to *Staphylococcus aureus*, with a total of 16 antibiotics detected out of 18 antibiotics tested, which is a high rate of detection of 89%, and the rest of the link also showed results of multi-resistance. *L. monocytogenes* was detected only in the hands of the X8 splitter and X9 splitter workers belonging to the splitting stage, and these two segments were multi-resistant and highly resistant to *L. monocytogenes*. Among the antibiotics banned by the Ministry of Agriculture, high levels of resistance were also detected, with enrofloxacin and ofloxacin being the most severe.

Although no pathogenic *E. coli* were detected, resistant contamination of *E*. *coli* remains a concern. The *E. coli* screened at X1, X4, and X8 sampling points showed multiple drug resistance and high drug resistance. It is worth noting that the *E. coli* screened at X1 and X6 sampling points were resistant to colistin to a high degree. *Salmonella* was only screened at X2, X3, and X6, but there were also cases of multiple drug resistance. In the antibiotic susceptibility test, ampicillin, tetracycline, flufenicol, enrofloxacin, ofloxacin and colistin are all veterinary drugs banned by the Ministry of Agriculture (2017. China) [52], but the susceptibility results show that the detected bacteria are still resistant. In the detection of Gram-positive bacteria *L. monocytogenes* and *Staphylococcus aureus*, *Staphylococcus aureus* can be detected at every step in the slaughtering and processing chain of broilers, and the resistance of *Staphylococcus aureus* in X6 exceptionally high, 16 antibiotics were detected in 18 antibiotics tested, the detection rate was as high as 89%, and other links also showed the results of multiple drug resistance. *L. monocytogenes* only detected in X8 and X9 links, which belong to the segmentation link. It also has multiple drug resistance and high drug resistance. Among the antibiotics banned by the Ministry of Agriculture, a high degree of resistance was also detected, with enrofloxacin and ofloxacin being the most severe. Overall, antimicrobial resistance showed an increasing trend along the slaughtering process. Based on the community analysis and the detection of drug-resistant food-borne pathogens, it is inferred that there is the possibility of cross-contamination in chicken slaughter and processing. Maintaining slaughter hygiene, regular microbiological monitoring of carcasses, implementing good production practices, and establishing a food safety system are necessary measures to minimize the risk to consumers and reduce the risk of food-borne pathogen infection [53]. Key points such as removal of internal organs and scalding and cooling points are refined and partitioned to prevent cross-infection [54].

## 4. Conclusions

In this study, we sampled ten key links in the production line of broiler slaughterhouses and qualitatively and quantitatively detected the ARGs associated with eight major classes of antibiotics and veterinary drugs banned by the Ministry of Agriculture, and the results showed that all types of antibiotic-associated ARGs were detected, among which tetracycline-resistant genes were detected at the highest rate, followed by macrolide-resistant genes and quinolone-resistant genes. The main ARGs and genes coding for actionable genetic factors detected in the broiler slaughter line included *sul2*, *tetX*, *floR*, *oqxB*, and *intI 1*. The highest abundance of ARGs and *intI 1* was detected in the scalding and cleaning processes. The highest abundance of *intI 1* was detected in the scalding session, suggesting that the contaminated ARGs in this session had a strong horizontal gene transfer ability; the highest abundance of ARGs in the gutting session was sul 2, suggesting that the ARGs in this session might come from gut microbial contamination due to offal extraction.

Microbial community structure correlated well with the sampling sessions. The scalding and offal picking sessions were representative of the structure of the two main types of microbiomes or the starting point of contamination, and the community compositions of the sessions following them were similar to these two sessions, respectively. *oqxB*’s potential host genus was *Enterobacteriaceae*. *Algae*, *Enterobacteriaceae*, and *Bacteroidetes* are potential host bacteria for *mefA*. The only *aac(6′)-ib-cr* potential host bacterium in *Delftobacteria*.

Foodborne pathogens were detected to varying degrees in all ten sampling sessions, with *Staphylococcus aureus* being the most prevalent; *Salmonella* was prevalent in sessions prior to gutting; *L. monocytogenes* was detected only on the surface of the parted meat and on the hands of the parted workers in the splitting session; and *C. jejuni* was detected very frequently in the scalded fading water and on the cleaned rifted meat. The results of drug sensitivity tests for *E. coli*, *S. aureus*, *Salmonella*, and *L. monocytogenes* detected at different stages of the process showed that most of them were highly resistant and multi-resistant. Among them, veterinary drugs that have been banned by the Ministry of Agriculture in China were detected in multiple links of drug resistance.

The main contamination of drug-resistant genes lies in the scalding water and gutting. It is recommended that slaughterhouses should reasonably handle the scalding water and replace it in time, and reasonably discharge it, and the gutting process should avoid scratching the viscera, which will cause drug-resistant contamination. The results preliminarily revealed the contamination of ARGs and ARBs in the slaughtering environment during broiler slaughtering. These data contribute to food safety risk assessment and the development of corresponding control measures. Although this study represents a survey of only one broiler slaughter-processing plant, it helps to provide information on the characteristics of the bacterial community in the chicken carcass and the contamination of ARGs and how it changes at various stages of processing. Similar surveys of multiple lines operating in different ways may help to better determine the nature and amount of contamination (especially pathogens) throughout the processing line. Future research will focus on developing alternatives to antibiotics for poultry farming, reducing the use of antibiotics and their accumulation in humans.

## Figures and Tables

**Table 1:** Information of samples collected.

Sample Name	Sampling Stage	Sampling Point	Number of Samples Collected
X1	scalding	carcasses surface	25
X2		cooling pool water	25
X3		carcasses surface after washing with cooling water	25
X4	disemboweling	carcasses surface	25
X5		knife surface	25
X6	eviscerating	entrails surface	25
X7	fragmenting	carcasses surface	25
X8		knife surface	25
X9		hand surface	25
X10		conveyor surface	25
	total	/	250

**Table 2:** Primers of qualitative detection.

Target Genes	Antibiotic	Primer Sequence (5′–3′)	Product Length (bp)
*tetA*	tetracyclines	GCGCGATCTGGTTCACTCG	164
		AGTCGACAGYRGCGCCGGC	
*tetC*	tetracyclines	CTTGAGAGCCTTCAACCCAG	418
		ATGGTCGTCATCTACCTGCC	
*tetG*	tetracyclines	GTCGATTACACGATTATGGC	432
		CACTTGGCCGATCAGTTGA	
*tetM*	tetracyclines	ACAGAAAGCTTATTATATAAC	171
		GGCGTGTCTATGATGTTCAC	
*tetO*	tetracyclines	ACGGARAGTTTATTGTATACC	171
		TGGCGTATCTATAATGTTGAC	
*tetQ*	tetracyclines	AGAATCTGCTGTTTGCCAGTG	169
		CGGAGTGTCAATGATATTGCA	
*tetT*	tetracyclines	AAGGTTTATTATATAAAAGTG	169
		AGGTGTATCTATGATATTTAC	
*tetW*	tetracyclines	AGGTGTATCTATGATATTTAC	168
		GGGCGTATCCACAATGTTAAC	
*tetX*	tetracyclines	GACCCGTTGGACTGACTATGG	168
		CTTCCTGACCTGAACCTTTGTG	
*sul1*	sulfonamides	CGCACCGGAAACATCGCTGCAC	163
		TGAAGTTCCGCCGCAAGGCTCG	
*sul2*	sulfonamides	TCCGGTGGAGGCCGGTATCTGG	191
		CGGGAATGCCATCTGCCTTGAG	
*Sul III*	sulfonamides	TCCGTTCAGCGAATTGGTGCAG	128
		TTCGTTCACGCCTTACACCAGC	
*ermB*	macrolides	CCGATACCGTTTACGAAATTGG	190
		TAGCAAACCCGTATTCCACG	
*mefA*	macrolides	AGTATCATTAATCACTAGTGC	186
		TTCTTCTGGTACTAAAAGTGG	
*bla_TEM_*	β-lactams	GCATCTTACGGATGGCATGA	99
		CCTCCGATCGTTGTCAGAAGT	
*bla_SHV_*	β-lactams	GGTTATGCGTTATATTCGCCTGTG	861
		TTAGCGTTGCCAGTGCTCGATCA	
*oqxA*	4-quinolones	GATCAGTCAGTGGGATAGTTT	627
		TACTCGGCGTTAACTGATTA	
*oqxB*	4-quinolones	TCCTGATCTCCATTAACGCCCA	131
		ACCGGAACCCATCTCGATGC	
*qnrD*	4-quinolones	ACGACAGGAATAGCTTGGAAGG	465
		TCAGCCAAAGACCAATCAAACG	
*floR*	Florfenicol	CGGTCGGTATTGTCTTCACG	171
		TCACGGGCCACGCTGTAT	
*aadA1*	aminoglycosides	AGCTAAGCGCGAACTGCAAT	195
		TGGCTCGAAGATACCTGCAA	
*aa* *c(6′)-ib-cr*	aminoglycosides	GCTCTATGAGTGGCTAAATCGATC	182
		GCAATGTATGGAGTGACGGAC	
*intI1*	class I integrons	CCTCCCGCACGATGATC	280
		TCCACGCATCGTCAGGC	
*mcr-1*	polymyxins	ATGATGCAGCATACTTCTGTG	1626
		TCAGCGGATGAATGCGGTG	
*mcr-2*	polymyxins	GATGGCGGTCTATCCTGTAT	715
		AAGGCTGACACCCCATGTCAT	
*mcr-3*	polymyxins	ACCAGTAAATCTGGTGGCGT	929
		AGGACAACCTCGTCATAGCA	
*mcr-4*	polymyxins	TTGCAGACGCCCATGGAATA	1116
		GCCGCATGAGCTAGTATCGT	
*mcr-5*	polymyxins	GGACGCGACTCCCTAACTTC	1644
		ACAACCAGTACGAGAGCACG	
*mcr-6*	polymyxins	GTCCGGTCAATCCCTATCTGT	556
		ATCACGGGATTGACATAGCTAC	
*mcr-7*	polymyxins	AGGGGATAAACCGACCCTGA	335
		TGATCTCGATGTTGGGCACC	
*mcr-8*	polymyxins	AACCGCCAGAGCACAGAATT	667
		TTCCCCCAGCGATTCTCCAT	
*mcr-9*	polymyxins	GGTAGTTATTCCGCTGG	1572
		TCGCGGTCAGGATTAAC	
*tetS*	tetracyclines	GGTCAACGGCTTGTCTATGTA	667
		CCAGGCTCTCATACTGAATGC	
*tetB*	tetracyclines	AAAACTTATTATATTATAGTG	169
		TGGAGTATCAATAATATTCAC	
*tetH*	tetracyclines	CAGTGAAAATTCACTGGCAAC	185
		ATCCAAAGTGTGGTTGAGAAT	
*qnrS*	4-quinolones	GCAAGTTCATTGAACAGGGT	428
		TCTAAACCGTCGAGTTCGGCG	
*qnrA*	4-quinolones	AGGATTTCTCACGCCAGGATT	124
		CCGCTTTCAATGAAACTGCAA	
*depA*	4-quinolones	CCAGCTCGGCAACTTGATAC	570
		ATGCTCGCCTTCCAGAAAA	
*gyrA*	4-quinolones	CAAGAATCGTGGGTGATG	351
		GTGGAATATTTGTCGCCA	
*ermC*	macrolides	GAAATCGGCTCAGGAAAAGG	293
		TAGCAAACCCGTATTCCACG	
*ermF*	macrolides	TCTAGCAATGAGAATGAAGGT	309
		ACTATAACGTGATGGTTGGGAGGGA	
*ermA*	macrolides	AAGCGGTAAACCCCTCTGA	190
		TTCGCAAATCCCTTCTCAAC	
*ereA*	macrolides	CCTTCACATCCGGATTCGCTCGA	420
		CTTCACATCCGGATTCGCTCGA	
*CatA1*	chloram phenicols	GGGTGAGTTTCACCAGTTTTGATT	101
		CACCTTGTCGCCTTGCGTATA	
*16S rDNA*	*-*	TGTGTAGCGGTGAAATGCG	140
		CATCGTTTACGGCGTGGAC	

## Data Availability

The original contributions presented in the study are included in the article, further inquiries can be directed to the corresponding authors.

## References

[1] WHO Antimicrobial Resistance: Global Report on Surveillance. https://www.who.int/publications/i/item/9789241564748.

[2] Fair R.J., Tor Y. (2014). Antibiotics and Bacterial Resistance in the 21st Century. Perspect. Med. Chem..

[3] Jeon J.H., Jang K.-M., Lee J.H., Kang L.-W., Lee S.H. (2023). Transmission of antibiotic resistance genes through mobile genetic elements in *Acinetobacter baumannii* and gene-transfer prevention. Sci. Total Environ..

[4] Zhang A.-N., Gaston J.M., Dai C.L., Zhao S., Poyet M., Groussin M., Yin X., Li L.-G., van Loosdrecht M.C.M., Topp E. (2021). An omics-based framework for assessing the health risk of antimicrobial resistance genes. Nat. Commun..

[5] Bai X., Zhong H., Cui X., Wang T., Gu Y., Li M., Miao X., Li J., Lu L., Xu W. (2024). Metagenomic profiling uncovers microbiota and antibiotic resistance patterns across human, chicken, pig fecal, and soil environments. Sci. Total Environ..

[6] Gao X.-L., Shao M.-F., Luo Y., Dong Y.-F., Ouyang F., Dong W.-Y., Li J. (2016). Airborne bacterial contaminations in typical Chinese wet market with live poultry trade. Sci. Total Environ..

[7] Theophilus R.J., Taft D.H. (2023). Antimicrobial Resistance Genes (ARGs), the Gut Microbiome, and Infant Nutrition. Nutrients.

[8] Ebmeyer S., Kristiansson E., Larsson D.G.J. (2021). A framework for identifying the recent origins of mobile antibiotic resistance genes. Commun. Biol..

[9] Peterson E., Kaur P. (2018). Antibiotic Resistance Mechanisms in Bacteria: Relationships Between Resistance Determinants of Antibiotic Producers, Environmental Bacteria, and Clinical Pathogens. Front. Microbiol..

[10] Fang H., Han L., Zhang H., Long Z., Cai L., Yu Y. (2018). Dissemination of antibiotic resistance genes and human pathogenic bacteria from a pig feedlot to the surrounding stream and agricultural soils. J. Hazard. Mater..

[11] Weinroth M.D., Thomas K.M., Doster E., Vikram A., Schmidt J.W., Arthur T.M., Wheeler T.L., Parker J.K., Hanes A.S., Alekoza N. (2022). Resistomes and microbiome of meat trimmings and colon content from culled cows raised in conventional and organic production systems. Anim. Microbiome.

[12] Liu Z., Klümper U., Shi L., Ye L., Li M. (2019). From Pig Breeding Environment to Subsequently Produced Pork: Comparative Analysis of Antibiotic Resistance Genes and Bacterial Community Composition. Front. Microbiol..

[13] Ben W., Wang J., Pan X., Qiang Z. (2017). Dissemination of antibiotic resistance genes and their potential removal by on-farm treatment processes in nine swine feedlots in Shandong Province, China. Chemosphere.

[14] Xu C., Kong L., Gao H., Cheng X., Wang X. (2022). A Review of Current Bacterial Resistance to Antibiotics in Food Animals. Front. Microbiol..

[15] Zhou Z.-C., Feng W.-Q., Han Y., Zheng J., Chen T., Wei Y.-Y., Gillings M., Zhu Y.-G., Chen H. (2018). Prevalence and transmission of antibiotic resistance and microbiota between humans and water environments. Environ. Int..

[16] Tu Z., Pang L., Lai S., Zhu Y., Wu Y., Zhou Q., Qi H., Zhang Y., Dong Y., Gan Y. (2024). The hidden threat: Comprehensive assessment of antibiotic and disinfectant resistance in commercial pig slaughterhouses. Sci. Total Environ..

[17] Carlet J. (2012). The gut is the epicentre of antibiotic resistance. Antimicrob. Resist. Infect. Control.

[18] Zhu Y., Lai H., Zou L., Yin S., Wang C., Han X., Xia X., Hu K., He L., Zhou K. (2017). Antimicrobial resistance and resistance genes in Salmonella strains isolated from broiler chickens along the slaughtering process in China. Int. J. Food Microbiol..

[19] Jia S., Zhang X.-X., Miao Y., Zhao Y., Ye L., Li B., Zhang T. (2017). Fate of antibiotic resistance genes and their associations with bacterial community in livestock breeding wastewater and its receiving river water. Water Res..

[20] Pu C., Yu Y., Diao J., Gong X., Li J., Sun Y. (2019). Exploring the persistence and spreading of antibiotic resistance from manure to biocompost, soils and vegetables. Sci. Total Environ..

[21] Wright G.D. (2010). Antibiotic resistance in the environment: A link to the clinic?. Curr. Opin. Microbiol..

[22] Liu J., Zhao Z., Avillan J.J., Call D.R., Davis M., Sischo W.M., Zhang A. (2019). Dairy farm soil presents distinct microbiota and varied prevalence of antibiotic resistance across housing areas. Environ. Pollut..

[23] Gaire T.N., Odland C., Zhang B., Slizovskiy I., Jorgenson B., Wehri T., Meneguzzi M., Wass B., Schuld J., Hanson D. (2024). Slaughtering processes impact microbial communities and antimicrobial resistance genes of pig carcasses. Sci. Total Environ..

[24] Franz E., van der Fels-Klerx H.J., Thissen J., van Asselt E.D. (2012). Farm and slaughterhouse characteristics affecting the occurrence of *Salmonella* and *Campylobacter* in the broiler supply chain. Poult. Sci..

[25] Statista Production of Poultry Meat Worldwide from 1990 to 2022. https://www.statista.com/statistics/237637/production-of-poultry-meat-worldwide-since-1990/.

[26] USDA Foreign Agricultural Service China—Poultry and Products Annual. https://fas.usda.gov/data/china-poultry-and-products-annual-7.

[27] Pan Y., Zeng J., Zhang L., Hu J., Hao H., Zeng Z., Li Y. (2024). The fate of antibiotics and antibiotic resistance genes in Large-Scale chicken farm Environments: Preliminary view of the performance of National veterinary Antimicrobial use reduction Action in Guangdong, China. Environ. Int..

[28] Yuan Y., Mo C., Huang F., Liao X., Yang Y. (2024). Microbial metabolism affects the antibiotic resistome in the intestine of laying hens. Poult. Sci..

[29] Temmerman R., Ghanbari M., Antonissen G., Schatzmayr G., Duchateau L., Haesebrouck F., Garmyn A., Devreese M. (2022). Dose-dependent impact of enrofloxacin on broiler chicken gut resistome is mitigated by synbiotic application. Front. Microbiol..

[30] Kpomasse C.C., Oke O.E., Houndonougbo F.M., Tona K. (2021). Broiler production challenges in the tropics: A review. Vet. Med. Sci..

[31] Chen S.H., Fegan N., Kocharunchitt C., Bowman J.P., Duffy L.L. (2020). Changes of the bacterial community diversity on chicken carcasses through an Australian poultry processing line. Food Microbiol..

[32] Nguyen A.Q., Vu H.P., Nguyen L.N., Wang Q., Djordjevic S.P., Donner E., Yin H., Nghiem L.D. (2021). Monitoring antibiotic resistance genes in wastewater treatment: Current strategies and future challenges. Sci. Total Environ..

[33] Tokuda M., Shintani M. (2024). Microbial evolution through horizontal gene transfer by mobile genetic elements. Microb. Biotechnol..

[34] Yan K., Wei M., Li F., Wu C., Yi S., Tian J., Liu Y., Lu H. (2023). Diffusion and enrichment of high-risk antibiotic resistance genes (ARGs) via the transmission chain (mulberry leave, guts and feces of silkworm, and soil) in an ecological restoration area of manganese mining, China: Role of heavy metals. Environ. Res..

[35] Lin Z., Yuan T., Zhou L., Cheng S., Qu X., Lu P., Feng Q. (2020). Impact factors of the accumulation, migration and spread of antibiotic resistance in the environment. Environ. Geochem. Health.

[36] Campos Calero G., Caballero Gómez N., Benomar N., Pérez Montoro B., Knapp C.W., Gálvez A., Abriouel H. (2018). Deciphering Resistome and Virulome Diversity in a Porcine Slaughterhouse and Pork Products Through Its Production Chain. Front. Microbiol..

[37] (2003). Microbiology Inspection of Food Hygiene Inspection of Meat and Meat Products.

[38] Scott G., Ryder D., Buckley M., Hill R., Treagus S., Stapleton T., Walker D.I., Lowther J., Batista F.M. (2024). Long Amplicon Nanopore Sequencing for Dual-Typing RdRp and VP1 Genes of Norovirus Genogroups I and II in Wastewater. Food Environ. Virol..

[39] (2016). Food Safety National Standard Food Microbiology Test *Listeria monocytogenes* Test.

[40] (2016). National Standard for Food Safety Microbiology Inspection of Food Salmonella Inspection.

[41] (2016). National Standard for Food Safety Food Microbiological Test *Staphylococcus aureus* Test.

[42] Zhang Y., Yang Z., Xiang Y., Xu R., Zheng Y., Lu Y., Jia M., Sun S., Cao J., Xiong W. (2020). Evolutions of antibiotic resistance genes (ARGs), class 1 integron-integrase (intI1) and potential hosts of ARGs during sludge anaerobic digestion with the iron nanoparticles addition. Sci. Total Environ..

[43] Yan W., Sun C., Zheng J., Wen C., Ji C., Zhang D., Chen Y., Hou Z., Yang N. (2019). Efficacy of Fecal Sampling as a Gut Proxy in the Study of Chicken Gut Microbiota. Front. Microbiol..

[44] Pinto Jimenez C.E., Keestra S., Tandon P., Cumming O., Pickering A.J., Moodley A., Chandler C.I.R. (2023). Biosecurity and water, sanitation, and hygiene (WASH) interventions in animal agricultural settings for reducing infection burden, antibiotic use, and antibiotic resistance: A One Health systematic review. Lancet Planet. Health.

[45] Wang Y., Dagan T. (2024). The evolution of antibiotic resistance islands occurs within the framework of plasmid lineages. Nat. Commun..

[46] Fang P., Peng F., Gao X., Xiao P., Yang J. (2019). Decoupling the Dynamics of Bacterial Taxonomy and Antibiotic Resistance Function in a Subtropical Urban Reservoir as Revealed by High-Frequency Sampling. Front. Microbiol..

[47] Jian Z., Zeng L., Xu T., Sun S., Yan S., Yang L., Huang Y., Jia J., Dou T. (2021). Antibiotic resistance genes in bacteria: Occurrence, spread, and control. J. Basic Microbiol..

[48] Conceição S., Queiroga M.C., Laranjo M. (2023). Antimicrobial Resistance in Bacteria from Meat and Meat Products: A One Health Perspective. Microorganisms.

[49] Cheng X., Lu Y., Song Y., Zhang R., ShangGuan X., Xu H., Liu C., Liu H. (2021). Analysis of Antibiotic Resistance Genes, Environmental Factors, and Microbial Community From Aquaculture Farms in Five Provinces, China. Front. Microbiol..

[50] Thames H.T., Fancher C.A., Colvin M.G., McAnally M., Tucker E., Zhang L., Kiess A.S., Dinh T.T.N., Sukumaran A.T. (2022). The Prevalence of *Salmonella* and *Campylobacter* on Broiler Meat at Different Stages of Commercial Poultry Processing. Animals.

[51] Boubendir S., Arsenault J., Quessy S., Thibodeau A., Fravalo P., Thériault W.P., Fournaise S., Gaucher M.-L. (2021). *Salmonella* Contamination of Broiler Chicken Carcasses at Critical Steps of the Slaughter Process and in the Environment of Two Slaughter Plants: Prevalence, Genetic Profiles, and Association with the Final Carcass Status. J. Food Prot..

[52] Ministry of Agriculture Announcement No. 2513, Ministry of Agriculture of the People’s Republic of China. https://www.moa.gov.cn/xw/bmdt/201704/t20170414_5560977.htm.

[53] Warmate D., Onarinde B.A. (2023). Food safety incidents in the red meat industry: A review of foodborne disease outbreaks linked to the consumption of red meat and its products, 1991 to 2021. Int. J. Food Microbiol..

[54] Pasquali F., Klaharn K., Pichpol D., Meeyam T., Harintharanon T., Lohaanukul P., Punyapornwithaya V. (2022). Bacterial contamination of chicken meat in slaughterhouses and the associated risk factors: A nationwide study in Thailand. PLoS ONE.

